# Supplementary material for: Optimal minimal residual disease threshold in pediatric acute myeloid leukemia: A retrospective cohort study based on the TARGET database
Source: PLoS Med. 2026 May 8;23(5):e1005088. doi: 10.1371/journal.pmed.1005088 (PMC13155632; doi:10.1371/journal.pmed.1005088)
Supplement: S1 Code — (ZIP) [file pmed.1005088.s002.zip › S2 code/PROJ8_8_tbl1/PROJ8_8_tbl1.htm]

## Cox Model

½á¾Ö±äÁ¿: First Event
Ê±¼ä±äÁ¿: EFS(years) 
 TIME.PHASE.4LMCOX : 1 
model:
Surv( X28 , X27 )~ X22

|  |  |
| --- | --- |
| ¹Û²ìÊý£¨ÈËÊý£© | 1205 |
| ÊÂ¼þ·¢ÉúÊý | 592 |

±ÈÀýÔÖº¦Ä£ÐÍ

|  |  |  |  |  |  |  |  |  |
| --- | --- | --- | --- | --- | --- | --- | --- | --- |
|  | »Ø¹éÏµÊý(coef) | »Ø¹éÏµÊý±ê×¼Îó | z | Î£ÏÕ±È(HR=exp(coef)) | 1/Î£ÏÕ±È | 95%Çø¼äÏÂÏÞ | 95%Çø¼äÉÏÏÞ | P¡¡Öµ |
| X22 | 1.0113 | 0.0865 | 11.6966 | 2.7493 | 0.3637 | 2.3207 | 3.2570 | <0.0001 |

 rsq = 0.0950, maxrsq = 0.9987
 Log Likelihood £¨¶ÔÊýËÆÈ»Öµ£©= -3954.23024416977

|  |  |  |  |
| --- | --- | --- | --- |
|  | test | df | pvalue |
| Likelihood ratio test | 120.34637 | 1.00000 | 0.00000 |
| Wald test | 136.81000 | 1.00000 | 0.00000 |
| Score(log rank)test | 148.67389 | 1.00000 | 0.00000 |

±ÈÀýÔÖº¦Ä£ÐÍÕï¶Ï

|  |  |  |  |
| --- | --- | --- | --- |
|  | rho | chisq | p |
| X22 | -0.1764 | 17.7088 | 0.0000 |

Somers' Rank Correlation for Censored Data

|  |  |  |  |  |  |  |  |
| --- | --- | --- | --- | --- | --- | --- | --- |
|  | C | Dxy | aDxy | SD | Z | P | n |
| Model | 0.6029 | 0.2058 | 0.2058 | 0.0186 | 11.073 | 0 | 1205 |

C: Harrell's C index; Dxy = 2(C - 0.5), Dxy is a U-statistic.
SD: Standard error of Dxy, so, SE. for C = SD/2 
 TIME.PHASE.4LMCOX : 2 
model:
Surv( X28 , X27 )~ X22

|  |  |
| --- | --- |
| ¹Û²ìÊý£¨ÈËÊý£© | 537 |
| ÊÂ¼þ·¢ÉúÊý | 19 |

±ÈÀýÔÖº¦Ä£ÐÍ

|  |  |  |  |  |  |  |  |  |
| --- | --- | --- | --- | --- | --- | --- | --- | --- |
|  | »Ø¹éÏµÊý(coef) | »Ø¹éÏµÊý±ê×¼Îó | z | Î£ÏÕ±È(HR=exp(coef)) | 1/Î£ÏÕ±È | 95%Çø¼äÏÂÏÞ | 95%Çø¼äÉÏÏÞ | P¡¡Öµ |
| X22 | 0.6296 | 0.5630 | 1.1183 | 1.8769 | 0.5328 | 0.6226 | 5.6584 | 0.2635 |

 rsq = 0.0020, maxrsq = 0.3293
 Log Likelihood £¨¶ÔÊýËÆÈ»Öµ£©= -106.681434302811

|  |  |  |  |
| --- | --- | --- | --- |
|  | test | df | pvalue |
| Likelihood ratio test | 1.10155 | 1.00000 | 0.29393 |
| Wald test | 1.25000 | 1.00000 | 0.26346 |
| Score(log rank)test | 1.29230 | 1.00000 | 0.25562 |

±ÈÀýÔÖº¦Ä£ÐÍÕï¶Ï

|  |  |  |  |
| --- | --- | --- | --- |
|  | rho | chisq | p |
| X22 | -0.2192 | 0.9196 | 0.3376 |

Somers' Rank Correlation for Censored Data

|  |  |  |  |  |  |  |  |
| --- | --- | --- | --- | --- | --- | --- | --- |
|  | C | Dxy | aDxy | SD | Z | P | n |
| Model | 0.5604 | 0.1208 | 0.1208 | 0.1094 | 1.1044 | 0.2694 | 537 |

C: Harrell's C index; Dxy = 2(C - 0.5), Dxy is a U-statistic.
SD: Standard error of Dxy, so, SE. for C = SD/2 
Total:
model:
Surv( X28 , X27 )~ X22 +factor( TIME.PHASE.4LMCOX )

|  |  |
| --- | --- |
| ¹Û²ìÊý£¨ÈËÊý£© | 1742 |
| ÊÂ¼þ·¢ÉúÊý | 611 |

±ÈÀýÔÖº¦Ä£ÐÍ

|  |  |  |  |  |  |  |  |  |
| --- | --- | --- | --- | --- | --- | --- | --- | --- |
|  | »Ø¹éÏµÊý(coef) | »Ø¹éÏµÊý±ê×¼Îó | z | Î£ÏÕ±È(HR=exp(coef)) | 1/Î£ÏÕ±È | 95%Çø¼äÏÂÏÞ | 95%Çø¼äÉÏÏÞ | P¡¡Öµ |
| X22 | 1.0016 | 0.0854 | 11.7320 | 2.7227 | 0.3673 | 2.3032 | 3.2187 | <0.0001 |
| factor(TIME.PHASE.4LMCOX)2 | -19.7902 | 1060.3076 | -0.0187 | 0.0000 | inf. | 0.0000 | Inf | 0.9851 |

 rsq = 0.3273, maxrsq = 0.9936
 Log Likelihood £¨¶ÔÊýËÆÈ»Öµ£©= -4061.15182129781

|  |  |  |  |
| --- | --- | --- | --- |
|  | test | df | pvalue |
| Likelihood ratio test | 690.53068 | 2.00000 | 0.00000 |
| Wald test | 137.64000 | 2.00000 | 0.00000 |
| Score(log rank)test | 526.73830 | 2.00000 | 0.00000 |

±ÈÀýÔÖº¦Ä£ÐÍÕï¶Ï

|  |  |  |  |
| --- | --- | --- | --- |
|  | rho | chisq | p |
| X22 | -0.1746 | 17.9227 | 0.0000 |
| factor(TIME.PHASE.4LMCOX)2 | -0.5763 | 0.0000 | 0.9998 |
| GLOBAL |  | 17.9227 | 0.0001 |

Somers' Rank Correlation for Censored Data

|  |  |  |  |  |  |  |  |
| --- | --- | --- | --- | --- | --- | --- | --- |
|  | C | Dxy | aDxy | SD | Z | P | n |
| Model | 0.7502 | 0.5004 | 0.5004 | 0.0149 | 33.566 | 0 | 1742 |

C: Harrell's C index; Dxy = 2(C - 0.5), Dxy is a U-statistic.
SD: Standard error of Dxy, so, SE. for C = SD/2 
Created by EmpowerStats (www.empowerstats.com) and R on 2025-10-07
